# Survival of *Borrelia burgdorferi* Strain B31 in Human Serum Is Not Dependent on C4BP Binding to the Bacterial Surface

**DOI:** 10.3390/pathogens13110976

**Published:** 2024-11-08

**Authors:** Tobias Jakobsson, Pär Comstedt, Sven Bergström, Johan Normark

**Affiliations:** 1 Department of Clinical Microbiology, Umeå University, 901 87 Umeå, Sweden; johan.normark@umu.se; 2 UCMR, Umeå Centre for Microbial Research, 901 87 Umeå, Sweden; sven.bergstrom@umu.se; 3 Evaxion Biotech, Dr. Neergaards vej 5F, 2970 Hørsholm, Denmark; pc@evaxion-biotech.com; 4 Department of Molecular Biology, Umeå University, 901 87 Umeå, Sweden

**Keywords:** *Borrelia*, C4b-binding protein, complement, serum survival

## Abstract

Lyme disease is a vector-borne illness caused by spirochetes belonging to the *Borrelia burgdorferi* species group. These bacteria employ several mechanisms to survive within the vertebrate host, including evasion of the complement system. In this study, we examine the protection against human serum killing by the binding of host complement regulators C4b-binding protein (C4BP) and factor H (FH) to the bacterial surface of *B. burgdorferi*. Via serum depletion of isolated complement regulators, we found that the absence of C4BP did not alter the survival of *B. burgdorferi* strain B31; however, the removal of FH increased the sensitivity of this strain to human serum as previously described. The *B. garinii* seabird-isolated strain Far04, on the other hand, did not bind any complement regulators of human origin and was serum-sensitive, indicating its special host species specificity.

## 1. Introduction

Lyme disease (LD) is a multisystemic infectious disease caused by spiral-shaped bacteria of the genus *Borrelia* [1]. The dissemination of the bacteria is dependent on blood meals from hard-bodied ticks, and during its life cycle, the bacteria encounter hosts of various species and is forced to adapt to changing immunological environments [2]. The capability to persist within hosts and thereby the host range has been shown to be largely, but not exclusively, dependent on the ability to survive in blood through resistance against the host complement system [3]. The complement system is a group of proteins that act in a cascade to clear pathogenic organisms. It achieves this through activating processes such as chemotaxis, enhancing phagocytosis, and lysing cells by forming a membrane attack complex [4]. Several mechanisms of complement evasion have been described in LD *Borrelia* species. These include the binding and inhibition of the early-stage classical pathway complement activator C1r via the bacterial lipoprotein BBK32, and the suppression of both the classical and lectin complement pathways by binding the complement subunit C4b via OspC [5]. The best-characterized and most-studied of the complement evasion mechanisms is the binding of the host complement regulatory protein factor H (FH) to the surface of the bacteria. Acquisition of FH and its truncated but functional form factor H-like protein 1 (FHL-1) on the bacterial surface enhances bacterial survival against serums from various vertebrate hosts, including humans, and several LD *Borrelia* proteins responsible for this action have been described and classified [6]. These include CspA, CspZ, and members of the OspE family. Members of the OspE family also bind a number of factor H-related proteins (CFHR). The binding of the other host complement regulator, C4b-binding protein (C4BP), to the surface of several genospecies of LD *Borrelia* have also been described [7]. C4BP is a serum-soluble protein complex that functions as an inhibitor of the classical and lectin pathways of the complement system, and the bacterial binding of C4BP as a virulence factor to enhance survival in human serum have been described in other bacterial pathogens with C4BP binding increasing serum survival in vitro of Nontypable *Haemophilus influenzae* and decreasing complement deposition on the surface of nonencapsulated pneumococci [8,9]. C4BP binds to a number of LD *Borrelia* species via an as of yet uncharacterized receptor but its relevance remains unexplored, raising the question: Does the binding of C4BP have an impact on survival, which could indicate this is a bacterial capability important for human infection? In this study, we examined the effect of C4BP removal from normal human serum (NHS) and the effect on serum survival of *B. burgdorferi* strain B31. Furthermore, we compare the effect on serum survival with *B. garinii* strain Far04 isolated from an *Ixodes uriae* tick feeding on Atlantic Puffin (*Fratercula arctica*).

## 2. Materials and Methods

### 2.1. Bacterial Strains and Culture Condition

Low-passage *B. burgdorferi* strain B31 (ATCC 35210) and *B. garinii* strain Far04 [10] were grown at 35 °C in a Barbour–Stoner–Kelly II (BSKII) medium supplemented with 6% rabbit serum (RS).

### 2.2. Preparation of Human Serum

Healthy individuals were recruited through the local blood bank (Umeå, Sweden). The requirement for an ethical permit was waived since all samples were, upon fully informed consent, anonymized and could not be traced back to the donors. Blood was allowed to clot for 20 min and normal human serum (NHS) was separated after 8 min of centrifugation at 5000 rpm at RT, followed by another centrifugation at 20,000 rpm for 5 min at RT. NHS not used directly was aliquoted and stored at −80 °C for further experiments. For subsequent experiments, NHS from at least five individuals were pooled. Heat-inactivated human serum (HIS) was prepared by incubating NHS at 56 °C for 30 min. After preparation, the HIS was stored at −20 °C. All sera were screened for the presence of anti-*Borrelia* antibodies by an in-house ELISA based on whole-cell proteins from *B. burgdorferi*, *B. afzelii*, and *B. garinii* prior to use (Supplementary Figure S5).

### 2.3. Serum Depletion Assay

Freshly prepared NHS was kept on ice and EDTA was added to a final concentration of 0.15 mM. Approximately 200 µg of monoclonal anti-C4BP or monoclonal anti-factor H IgG (Santa Cruz Biotechnology, Dallas, TX, USA) was coupled to AB SpinTrap HP Protein G-sepharose columns (GE Healthcare Lifesciences, Cardiff, UK). Sera were added to the columns and incubated at 4 °C for 30 min under gentle agitation and collected by centrifugation. The complete removal of C4BP and FH was verified by Western blot (Supplementary Figures S3 and S4). Control serum, NHS, and HIS were passed through columns without coupled antibodies. The retained complement activity in the depleted serum and control serum was verified by erythrocyte lysis assay as previously described [11] (Supplementary Figure S6) and WIESLAB Complement System Screen ELISA kit (Supplementary Figure S7) (Euro Diagnostica, Malmö, Sweden).

### 2.4. Serum Survival Assay

*Borrelia* strains B31 and Far04 were grown to a density of 10^7^ spirochetes/mL, as determined using a Petroff-Hausser counting chamber. Spirochetes from the same cultivation batch were used for both assessing serum survival and the capability to bind complement regulators. To determine serum survival, 10^5^ spirochetes in a 10 µL BSKII medium were added to 90 µL 50% NHS, depleted serum, or HIS (diluted in PBS). After the addition of bacteria, the mixture was supplemented with 0.4 mM CaCl_2_ and 0.7 mM MgCl_2_, and incubated for 1 h at 37 °C under light agitation. All samples were tested in quadruplicates. Twenty microliters (20 µL) of each spirochete/serum suspension were added to a 180 µL BSKII medium supplemented with 6% RS in a 96-well microtiter plate. The plate was sealed with Parafilm (Sigma-Aldrich, St. Louis, MO, USA) and incubated for 9 days at 35 °C. Spirochete growth was assessed by microscopy examination and daily measurements of absorbance at 560 nm. As spirochetes multiplied, their acidic metabolic products caused the pH indicator phenol red in the BSKII medium to change from orange to yellow. This color change correlated with increased bacterial density, as indicated by decreased absorbance. To detect possible contamination and assure spirochete viability, one well per sample was assessed for bacterial growth non-quantitatively on days 3, 5, and 9 using Zeiss Axioscope 40 Phase Contrast Microscope (Zeiss, Oberkochen, Germany) at 400 times magnification.

### 2.5. Complement Binding Assay

The capability of B31 and Far04 to bind serum-soluble complement regulators was evaluated as previously described [7]. In short, 5 × 10^7^ spirochetes were washed five times in an in-house-prepared veronal buffered saline (VBS) and incubated in 50% HIS (diluted in PBS 20 mM EDTA) for 60 min at 37 °C under light agitation. Serum-absorbed spirochetes were washed five times in VBS and depleted of bound proteins in 0.1 M glycine-HCl, pH 2 for 15 min at RT. After elution, the samples were centrifuged at 8000 rpm for 4 min at RT and the supernatants were collected.

Eluted proteins were subjected to SDS-PAGE under non-reducing conditions (NuPAGE-Novex, Life technologies, Carlsbad, CA, USA) and transferred to PVDF membranes (Pall Corporation, Port Washington, NY, USA). Membranes were blocked in fat-free milk and incubated with monoclonal anti-human FH or C4BP antibodies (both at dilution 1:500) (Santa Cruz Biotechnology, Dallas, TX, USA) and signal was detected via primary, corresponding HRP-conjugated secondary antibodies using the Amersham ECL Western blot detection kit according to the manufacturer’s instructions (GE Healthcare Lifesciences, Cardiff, UK) and visualized with a Fujifilm LAS 4000 imaging system (GE Healthcare Lifesciences, Cardiff, UK). Factor H and C4BP procured commercially were used as positive controls (Complement Technology, Tyler, TX, USA).

## 3. Results and Discussion

**Figure 1:**
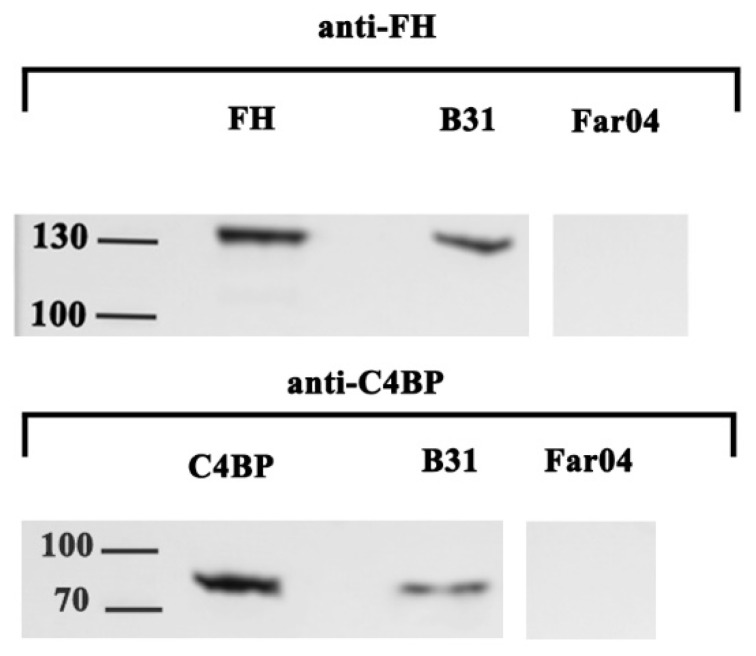
Western blot detection of FH and C4BP in eluates from serum-absorbed bacteria. FH and C4BP proteins were used as positive control (full blots, Supplementary Figures S1 and S2).

**Figure 2:**
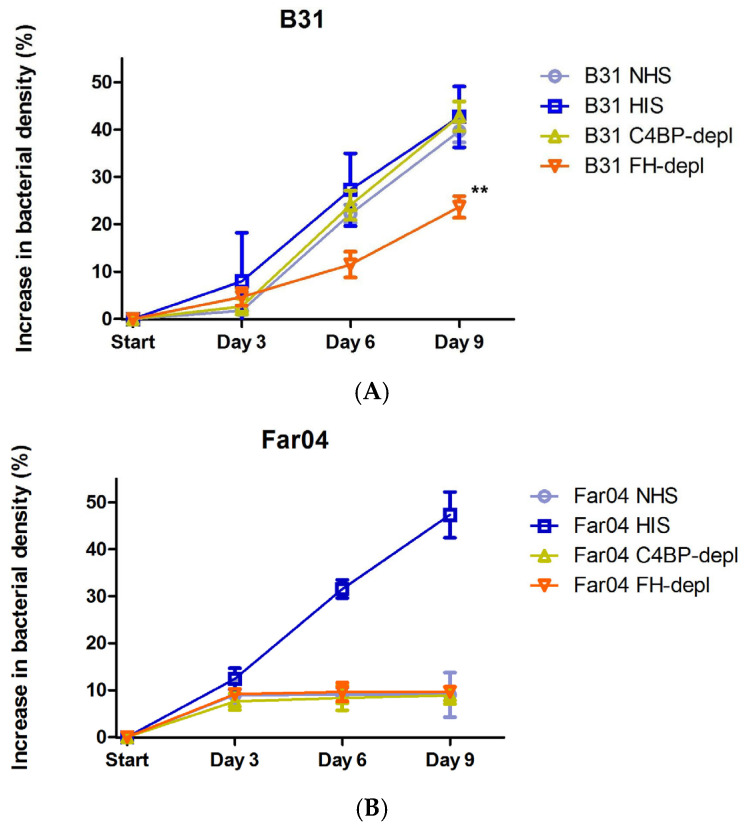
Growth curves of strain B31 (**A**) and Far04 (**B**) after incubation in NHS, HIS, and serum depleted of complement regulators. Mean increases in bacterial growth with 95% CI are shown. Statistical analysis was performed using one-way ANOVA followed by Dunnett’s post hoc test where appropriate, with growth in NHS serving as control. Significant differences marked with ** (*p*-value < 0.01).

In this study, we examined if removal of C4BP from NHS, and subsequent abolished binding of the complement regulator to the surface of the *B. burgdorferi* strain B31 produced a measurable difference in serum survival in vitro. Previous studies have employed serum depletion of C4BP [12,13] and FH [14,15] to investigate the protective effect of these regulators’ binding to the surface of various bacterial species. Binding C4BP to the bacterial surface theoretically protects against the classical and lectin pathways of the complement system. This is achieved by preventing complement factor C4b from attaching to the bacterial cell membrane. Without this attachment, the formation of the classical/lectin pathway C3-convertase is hindered, and downstream complement enzymatic steps are not activated, preventing bacterial lysis. The binding of C4BP could also accelerate the degradation of formed C3 convertase [4]. Both the classical and lectin pathways have been shown to be important in the clearance of *Borrelia* infections in murine in vivo systems [16,17], and reduced activity levels in the lectin pathway have been suggested to be a risk factor for *Borrelia* infection in humans [18]. The serum-resistant strain B31 bound both FH and C4BP of human origin to its surface when incubated in HIS, whereas the serum-sensitive strain Far04 bound neither of the two serum complement regulators (Figure 1). To assess the impact on serum survivability in the absence of C4BP and FH, bacteria were incubated in FH- or C4BP-depleted NHS with inhibitory levels of EDTA. The samples were then reconstituted with MgCl_2_ and CaCl_2_ to restore complement activity. Depletion of C4BP from NHS did not alter serum survival of the strain B31 as the bacteria showed the same increase in bacterial density as after incubation in NHS or HIS. Removal of FH from NHS did result in a measurable effect on the growth of B31 with a significant reduction in the observed bacterial density (Figure 2). However, the removal of FH did not render the bacteria serum-sensitive. Instead, bacterial density increased, although at a lower rate than the non-depleted control. Motile spirochetes were observed via microscopy at the final time point on day 9. Strain Far04’s serum survival remained unchanged in FH- or C4BP-depleted serum, exhibiting the same growth inhibition pattern as when challenged with NHS (Figure 2). Thus, in our study, the removal of C4BP did not alter the capability of the serum-resistant strain B31 to survive in human serum, indicating this capability to likely be non-essential. Redundancy and multifunctionality in terms of complement interaction in *Borrelia* is well described and mechanisms in LD *Borrelia* that affect the classical and lectin pathways without C4BP include BBK32s inhibition of C1r and the binding of C4b to OspC [19]. Exposing B31 to FH-depleted NHS showed a measurable effect on bacterial growth, supporting the protective role of FH bound to the surface of LD *Borrelia*. However, in the absence of FH, the bacteria were not killed by NHS but rather experienced stunted growth. The antibodies used to make FH-depleted serum in this study were raised against full length FH, and displayed no reactivity towards FHL-1 or any CFHR. The continued presence of this/these protein(s) in the serum can therefore not be excluded as a reason as to why B31 displayed an intermediate serum sensitivity rather than becoming fully sensitive in the absence of FH. The *B. garinii* strain Far04 was even more sensitive to human serum, since it showed no binding of complement regulators and retained its serum sensitivity when FH and C4BP were removed. The sensitivity to human serum is likely explained by its avian origin and highlights the theory that certain *B. garinii* strains have adapted to a narrower reservoir niche [20]. Far04 lacks the cp32 plasmids and its complement regulatory Erp protein family but possesses homologues of the *cspZ* gene and lp54 plasmid with its corresponding PFam54 gene array, which is involved in complement interactions in other *Borrelia* species [20,21]. The inability to bind FH of human origin observed in this study correlates with previous findings described for other *B. garinii* isolates [22,23].

Experiments in vitro with complement protein interactions is error-prone, as the handling of the serum itself may trigger the complement cascade. This may cause the consumption of individual subfactors and thus mandates strict control experiments [24]. In this study, we employed a commercially available ELISA-based kit that measures neoantigens formed upon activation of selected pathways to all sera. As this test measures the final products of complement activation and might display accidentally activated complement as functional, we also used an erythrocyte lysis assay that compared the relative hemolysis of the classical and alternative pathways to a reference mean hemolysis of all tested sera before pooling. We observed a slight loss of activity in the alternative pathway after pooling but the hemolytic activity of the sera did not change further during the experiments. The column-passed and depleted sera lysed erythrocytes to a similar grade as pooled NHS when MgCl_2_ and CaCl_2_ were added. As the depletion of FH from serum is especially known to cause rapid activation of the alternative pathway if the reaction is not hindered by chelating chemicals [25], one could argue that this does not correspond to a physiological activation in the presence of bacteria, despite the retained ability to lyse erythrocytes. It is therefore not excluded that the alternative pathway’s changed reaction pattern in FH-depleted serum might have influenced serum survival in this part of the study. In this study, we applied several control experiments to validate the serum potency and the serum-sensitive strain Far04 remained sensitive to both C4BP- and FH-depleted sera. In summary, the removal of C4BP did not alter survival of the B31 strain. This is interesting as this shows that C4BP binding as a singular factor does not explain B31’s capability to survive in the presence of human complement. LD *Borrelia* have developed several mechanisms to counter the various pathways of the complement system at different stages of the cascade and this display of multifunctionality and redundancy indicates that complement survival of this pathogen is not a one-factor event but rather the sum of many mechanisms. These findings show that further studies are needed that determine the roles that these individual mechanisms play alone and in conjuncture with each other in LD *Borrelia* complement survival.

## Data Availability

The original contributions presented in the study are included in the article/Supplementary Materials; further inquiries can be directed to the corresponding author.

## Supplementary Materials

The following supporting information can be downloaded at: https://www.mdpi.com/article/10.3390/pathogens13110976/s1, Figure S1: FH-absorbed full blot. Figure S2: C4BP-absorbed full blot. Figure S3: FH blot-depleted serum. Figure S4: C4BP blot-depleted serum. Figure S5: Serology ELISA results. Figure S6: Erythrocyte lysis results. Figure S7: Complement function ELISA results.
