# Supplementary figures and images for: Survival of Borrelia burgdorferi Strain B31 in Human Serum Is Not Dependent on C4BP Binding to the Bacterial Surface
Source: Pathogens. 2024 Nov 8;13(11):976. doi: 10.3390/pathogens13110976 (PMC11597344; doi:10.3390/pathogens13110976)

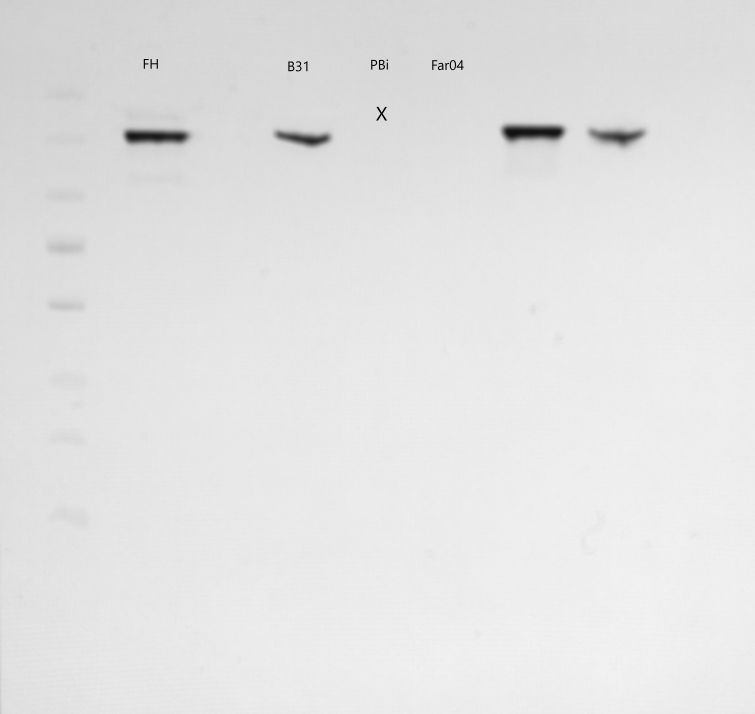

Supplement: Supplementary file 1 [file pathogens-13-00976-s001.zip › S1 FH adsorbed full blot.jpg]

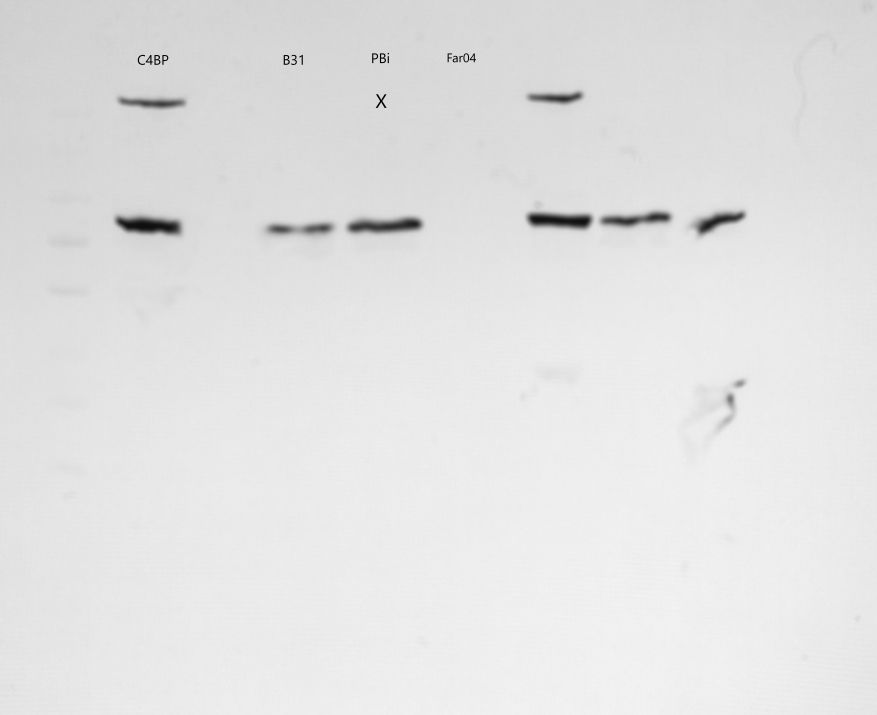

Supplement: Supplementary file 1 [file pathogens-13-00976-s001.zip › S2 c4bp adsorbed full blot.jpg]

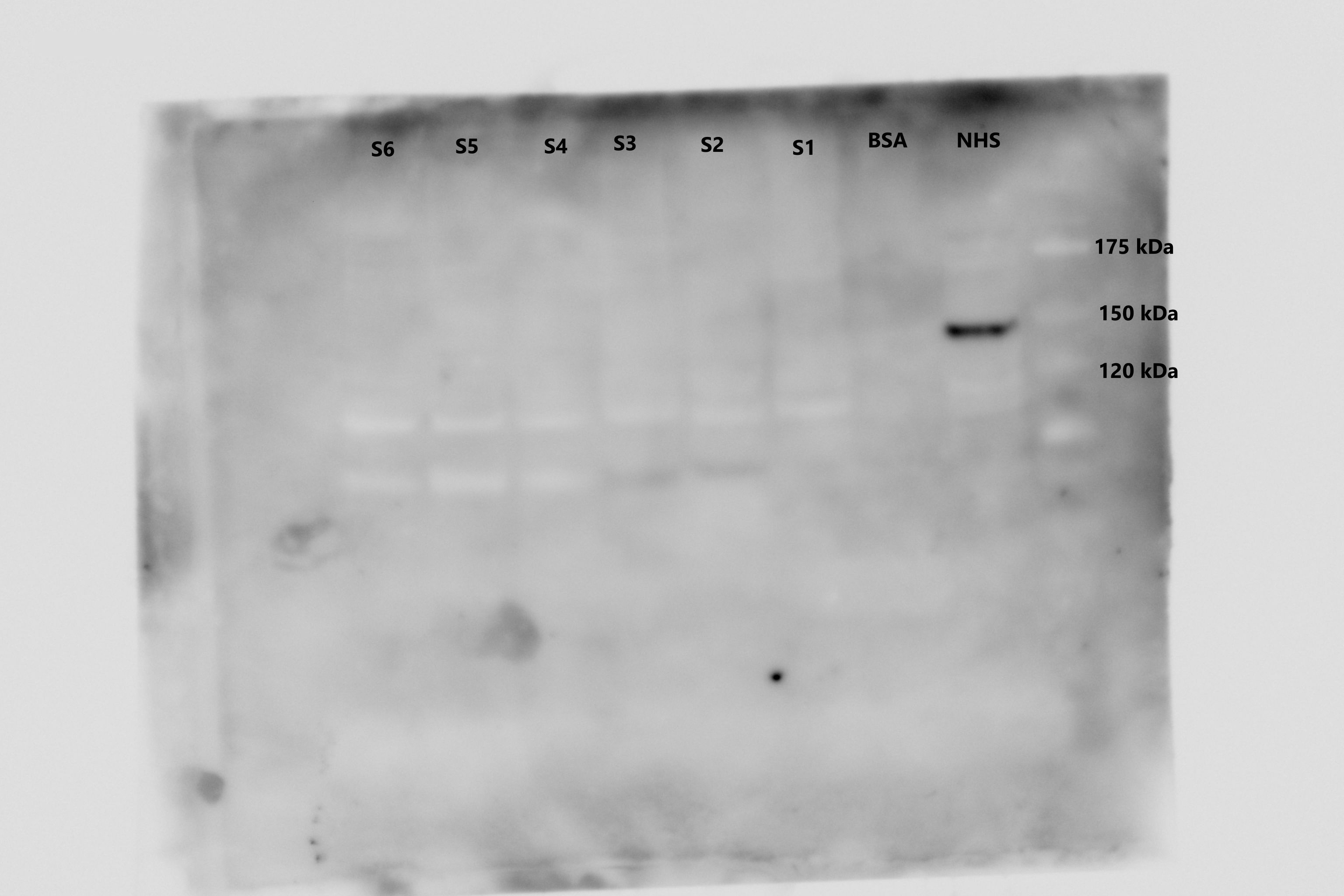

Supplement: Supplementary file 1 [file pathogens-13-00976-s001.zip › S3 FH blot depleted serum.jpg]

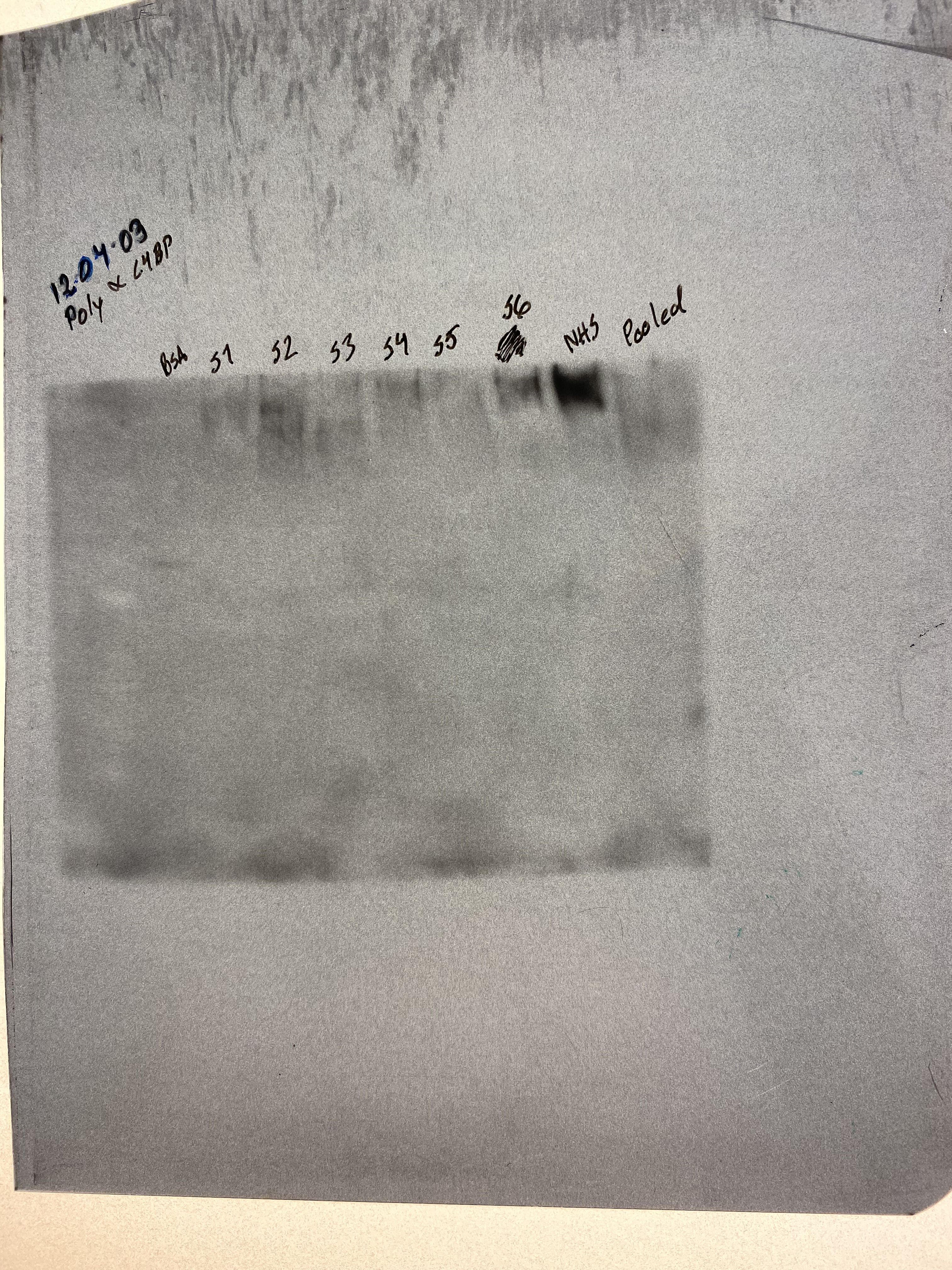

Supplement: Supplementary file 1 [file pathogens-13-00976-s001.zip › S4 C4BP blot depleted serum.jpg]

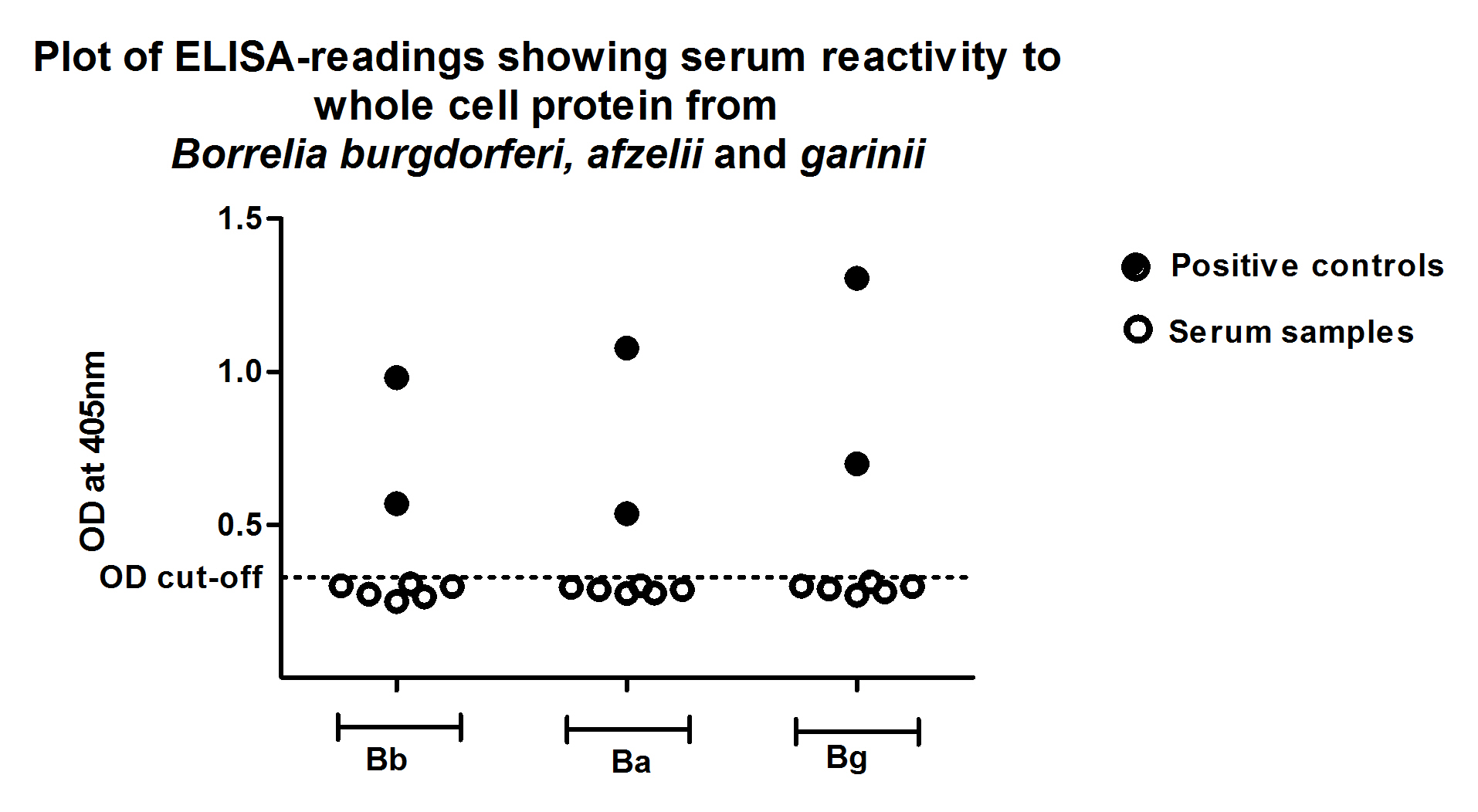

Supplement: Supplementary file 1 [file pathogens-13-00976-s001.zip › S5 Serology ELISA results.jpg]

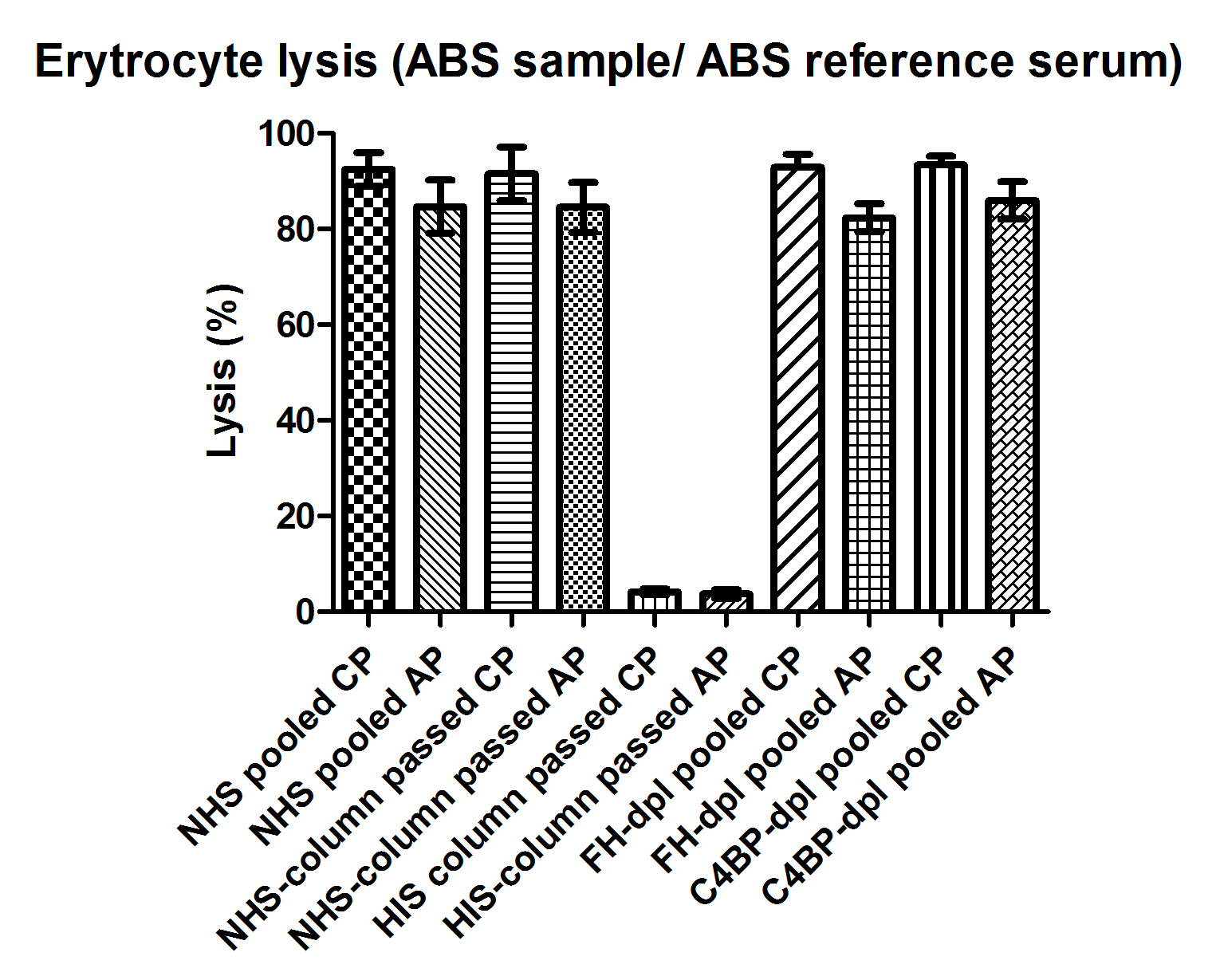

Supplement: Supplementary file 1 [file pathogens-13-00976-s001.zip › S6 Erytrocyte lysis results.jpg]

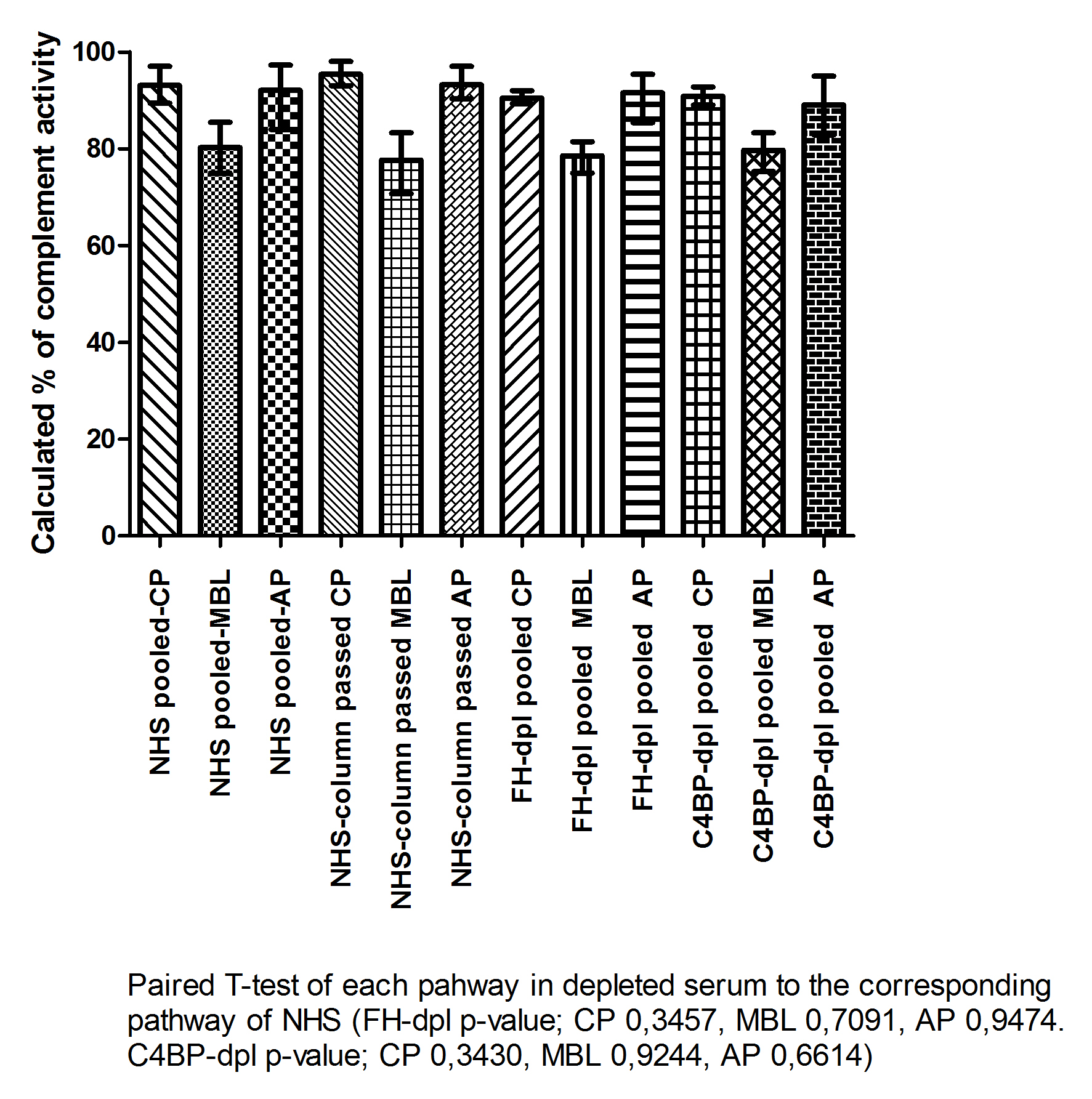

Supplement: Supplementary file 1 [file pathogens-13-00976-s001.zip › S7 Complement function ELISA results.jpg]
